# Patients with Tuberculosis Have a Dysfunctional Circulating B-Cell Compartment, Which Normalizes following Successful Treatment

**DOI:** 10.1371/journal.ppat.1005687

**Published:** 2016-06-15

**Authors:** Simone A. Joosten, Krista E. van Meijgaarden, Franca del Nonno, Andrea Baiocchini, Linda Petrone, Valentina Vanini, Hermelijn H. Smits, Fabrizio Palmieri, Delia Goletti, Tom H. M. Ottenhoff

**Affiliations:** 1 Department of Infectious Diseases, Leiden University Medical Center, Leiden, The Netherlands; 2 Pathology Service, National Institute for Infectious Diseases, Rome, Italy; 3 Department of Epidemiology and Preclinical Research, National Institute for Infectious Diseases, Rome, Italy; 4 Department of Parasitology, Leiden University Medical Center, Leiden, The Netherlands; 5 Clinical Department, National Institute for Infectious Diseases, Rome, Italy; Portland VA Medical Center, Oregon Health and Science University, UNITED STATES

## Abstract

B-cells not only produce immunoglobulins and present antigens to T-cells, but also additional key roles in the immune system. Current knowledge on the role of B-cells in infections caused by intracellular bacteria is fragmentary and contradictory. We therefore analysed the phenotypical and functional properties of B-cells during infection and disease caused by *Mycobacterium tuberculosis* (Mtb), the bacillus causing tuberculosis (TB), and included individuals with latent TB infection (LTBI), active TB, individuals treated successfully for TB, and healthy controls. Patients with active or treated TB disease had an increased proportion of antibodies reactive with mycobacteria. Patients with active TB had reduced circulating B-cell frequencies, whereas only minor increases in B-cells were detected in the lungs of individuals deceased from TB. Both active TB patients and individuals with LTBI had increased relative fractions of B-cells with an atypical phenotype. Importantly, these B-cells displayed impaired proliferation, immunoglobulin- and cytokine- production. These defects disappeared upon successful treatment. Moreover, T-cell activity was strongest in individuals successfully treated for TB, compared to active TB patients and LTBI subjects, and was dependent on the presence of functionally competent B-cells as shown by cellular depletion experiments. Thus, our results reveal that general B-cell function is impaired during active TB and LTBI, and that this B-cell dysfunction compromises cellular host immunity during Mtb infection. These new insights may provide novel strategies for correcting Mtb infection-induced immune dysfunction towards restored protective immunity.

## Author Summary

In infections with intracellular pathogens like *Mycobacterium tuberculosis* (Mtb), B-cells have long been ignored as their primary product, immunoglobulins, are unlikely to recognize intracellular bacteria. However, we have analysed here the frequency, phenotype and function of B-cells in tuberculosis (TB) infection and disease. Our data revealed that during active TB disease B-cell numbers are decreased and remaining B-cells are functionally impaired. Surprisingly, also individuals recently infected with Mtb suffered from poorly functional B-cells, but patients cured from the disease recovered with normal B-cell numbers and function. Thus, B-cell dysfunction contributes to impaired immune activation during Mtb infection.

## Introduction

Human B-cells not only mediate humoral immunity but are also key players in the initiation and regulation of T-cell responses. B-cells can act as professional antigen presenting cells, provide co-stimulatory signals, produce cytokines and can exert immunoregulatory properties. Antigen uptake by B-cells typically occurs via the B-cell-receptor; however, live mycobacteria can also infect B-cells through macropinocytosis, resulting in MHC class II antigen presentation [1–3]. Although less appreciated, B-cells exist in multiple flavours, not unlike the large variety of T-cell subsets. By implication, the type of B-cell that activates T-cells may critically determine the final fate and direction of the ensuing T-cell response. B-cells can be divided into subpopulations based on lineage and differentiation markers, and include naïve B-cells, immature B-cells, plasma cells, regulatory B-cells (Bregs) and memory B-cells [4]. Memory B-cells can be further subdivided into classical, active and atypical B-cells, based on the combined expression patterns of CD21 and CD27 or IgD and CD27 [5,6].

The role of B-cells in infectious diseases, in particular intracellular bacterial infections such as with *Mycobacterium tuberculosis* (Mtb) has not been investigated in great detail, mostly because B-cell derived immunoglobulins were considered not to play a prominent role in infections with intracellular pathogens [7]. However, B-cells have lately been rehabilitated as important players in the immune response during chronic inflammation irrespective of immunoglobulin production. Nevertheless, in human TB B-cell phenotypes and function have not been extensively investigated.

Studies enumerating B-cells in patients with TB disease have yielded conflicting results, not only in patients with active pulmonary TB but also in latently TB infected individuals (LTBI). In active TB, B-cell frequencies have been reported to be unaltered [8]; increased [9]; or decreased [10,11] compared to healthy controls. In addition, compared to healthy donors, LTBI individuals have been reported to have decreased B-cell frequencies [10], whereas those successfully treated for TB had increased B-cell frequencies [8]. In addition, patients with multi-drug resistant (MDR) TB were reported to have decreased frequencies of unswitched, IgD^+^CD27^+^ B-cells and decreased plasma cell frequencies, which are frequently observed during chronic inflammation [12]. The results described so far are rather conflicting and highly descriptive, without any analyses of the functional capacities of the B-cells. The only functional assessment of B-cells was described for a very small group of only 3 TB patients, which suggested hampered proliferation of B-cells following specific antigenic stimulation but did not take into account absolute B-cell numbers or phenotypes [13].

The contribution of B-cells to TB disease development has been studied in animal infection models, mostly in mice with genetic B-cell deficiencies. Infection of B-cell deficient mice with virulent Mtb resulted in enhanced pathology and increased bacterial loads [14,15], depending on the route of infection, either the lung [15] or systemic [14]. Moreover, these models showed less severe granuloma formation in the lungs and delayed dissemination of bacteria, indicating a role for B-cells in coordination of granuloma formation [15–17]. Indeed, transfer of B-cells, but not immunoglobulins, restored granuloma formation and inflammation [17]. However, not all studies revealed the same phenotypes, including increased bacterial loads, in B-cell and IL-4 deficient mice. Recently, B-cell depletion studies were performed in Mtb infected non-human primates and although disease severity and clinical outcome were not altered, local inflammation and bacterial loads appeared to be critically modulated by B-cells [18]. Cytokines like T-cell derived IL-17, IL-2 and IL-10 are increased in granuloma’s, whereas IL-6 and IL-10 are decreased [18].

Analysis of murine lungs during Mtb infection revealed an 8-fold increase in the absolute number of B-cells (CD19^+^) in the lung, representing 6–8% of CD45^+^ leucocytes (normally 2%), compared to very early stages of infection. These levels remained persistently elevated during chronic Mtb infection [19]. Histological analysis of the lungs showed a strong clustering of B-cells together with dendritic cells (DCs), surrounded by T-cells [19]. These clusters were also observed in human lungs affected by TB disease and recognized as germinal-center like structures [15,16,20]. Characterization of these clusters suggested lymphoneogenic formation of tertiary lymphoid nodules at the site of disease. B-cells in these ectopic B-cell follicles expressed GL7 (a classical B-cell germinal center marker) and CXCL13 (strongly associated with lymphoid neogenesis) [15].

In addition to these animal and histological studies, further indications for a potential involvement of B-cells in TB have emerged from unbiased studies. Searches for host biomarkers of TB disease and of curative responses to TB treatment in blood samples revealed decreased mRNA expression of B-cell related genes during active TB disease, which increased again following treatment [21,22]. B-cell associated genes were among the strongest differentially regulated genes between time points of diagnosis and end of treatment [21]. Moreover, combined analysis of gene expression data from 8 independent studies also revealed strongly altered expression of B-cell related genes, suggesting that B-cells are significantly involved in TB disease [23]. In another recent study we identified IL13 mRNA expression, a B-cell promoting cytokine, months before actual TB diagnosis as a correlate of risk for the development of TB disease in HIV infected individuals [24].

Therefore, to better understand the role of B-cells during Mtb infection we have analysed B-cell phenotypes and functional properties in peripheral blood cells from patients at different stages of TB infection and disease. We also analysed in situ B cells in lung specimens from deceased TB patients. We find unexpectedly large differences between patients with active TB and controls, indicating impaired B-cell functions in active TB patients. Unexpectedly also individuals with LTBI showed reduced B-cell functionalities. Moreover, our findings demonstrate that normally functioning B-cells, which are present in treated TB patients, are important for optimal activation of human Mtb-specific T-cell immunity.

## Results

Twenty-two individuals with LTBI were compared to 23 patients with active pulmonary TB (TB) and 27 individuals that had successfully completed treatment for pulmonary TB (TB treated) (Table 1). As uninfected healthy controls, 18 subjects from INMI and 10 subjects from the Dutch blood bank cohort were used. All groups included individuals with mixed ethnic backgrounds. Many from the Italian cohort had previously received the BCG vaccine. In all groups about 60% were females, except the group with active TB which comprised of only 13% females. Controls were largely from Western European origin whereas TB cases where mostly of Eastern European origin. LTBI and treated TB groups had similarly mixed Eastern and Western European descend. The group of LTBI individuals were mainly family members of TB patients (15/22) recruited at diagnosis of TB index cases (not necessarily the TB patients assessed here), thus all very shortly after documented Mtb exposure, however not excluding previous exposure and/ or infection or re-infection.

**Table 1:** Demographic and clinical information of the patients enrolled for the study.

		controls	LTBI	Active TB	TB treated
**N**		18	22	23	27
**Age median (IQR)**		38 (25–57)	38 (21–77)	42 (23–73)	40 (18–70)
**Gender Female**		11 (61)	15 (68)	3 (13)	16 (59)
**BCG-vaccinated**		6 (33)	9 (41)	19 (82)	17 (63)
**Months post treatment median (IQR)**		-	-	-	8 (1–72)
**Origin, N (%)**					
	**West Europe**	15 (83)	13 (59)	4 (17)	10 (37)
	**Est Europe**	2 (11)	5 (23)	15 (65)	9 (33)
	**Asia**	-	-	1 (4)	2 (7)
	**Africa**	-	2 (9)	2 (9)	3 (11)
	**Sud America**	1 (6)	2 (9)	1 (4)	3 (11)
**Cavities, N (%)**					
	**0**	-	-	8 (35)	-
	**1**	-	-	8 (35)	-
	**2**	-	-	1 (4)	-
	**>2**	-	-	6 (26)	-
**Number of lungs involved** * **, N (%)**					
	**1 lung**			13 (57)	
	**2 lungs**			10 (43)	
**First line drug sensitive, N (%)**					
	**Yes**	-	-	23 (100)	27 (100)
	**No**	-	-	0	0
**TB treatment outcome** ** **, N (%)**					
	**Successful**	-	-	23 (100)	27 (100)
	**No successful**	-	-	0	0
**AFB score in the smear, N (%)**					
**score**	**0**	-	-	4 (17)	-
	**1**	-	-	3 (13)	-
	**2**	-	-	6 (26)	-
	**3**	-	-	10 (44)	-

TB: tuberculosis; AFB: Acid Fast Bacilli; LTBI: latent TB infection; BCG: Bacillus Calmette et Guerin; IQR: Interquartile Range

*all lesions: cavities, nodular lesions, consolidation

**culture negative at 2 and 6 months after therapy.

### Increased antibody levels reactive with PPD in patients with active TB and treated TB patients compared to controls

**Fig 1:**
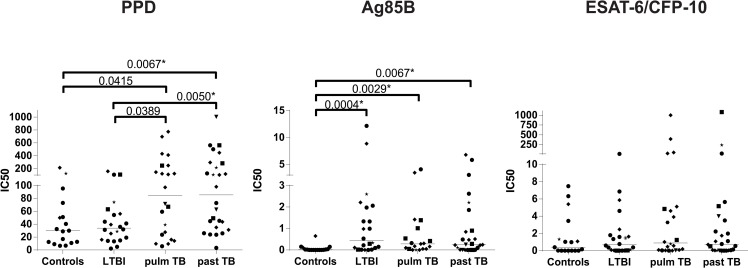
Antibodies reactive with PPD are present in active and treated TB patients. Plasma samples of from individuals infected with Mtb, with active TB disease or successfully treated TB patients and controls were tested by ELISA for the presence of IgG antibodies reactive with PPD (left), Ag85B (middle) and ESAT-6/CFP-10 (right). Ethnicity of donors was indicated using the following symbols: ‘black circle’ = West Europe; ‘black diamond’ = Est Europe; ‘black square’ = Africa; ‘black triangle’ = Asia; ‘black star’ = Sud America. Groups were compared to the uninfected controls using the Mann-Whitney test and a p< 0.05 was considered significant. * marks differences that remained significant after multiple test correction using Kruskal-Wallis testing with Dunn’s post-test.

Plasma samples from Italian controls, individuals with latent Mtb infection, active or treated TB disease were tested for IgG antibodies reactive with PPD, Ag85B or ESAT-6/CFP-10. We detected IgG antibodies against PPD in patients with active TB disease and those successfully treated for TB at a higher level compared to LTBI or control individuals (Fig 1). Antibodies reactive with Ag85B were detected in all infected groups compared to controls, albeit at very low levels (Fig 1). ESAT-6/ CFP-10 specific antibodies were detected in some individuals at a very high level, however no differences were observed between the groups (Fig 1).

### B-cell frequencies are decreased, and the relative fraction of B-cells with an atypical phenotype is increased in active TB

We analysed individuals with latent Mtb infection, active or treated TB disease and compared those to healthy uninfected controls.

Patients with active TB had significantly increased numbers of leucocytes in their peripheral blood, mostly as a result of increased numbers of circulating neutrophils and monocytes (S1A Fig). The percentage of lymphocytes showed a relative decrease at the time of diagnosis, although the absolute number remained constant (S1B Fig).

**Fig 2:**
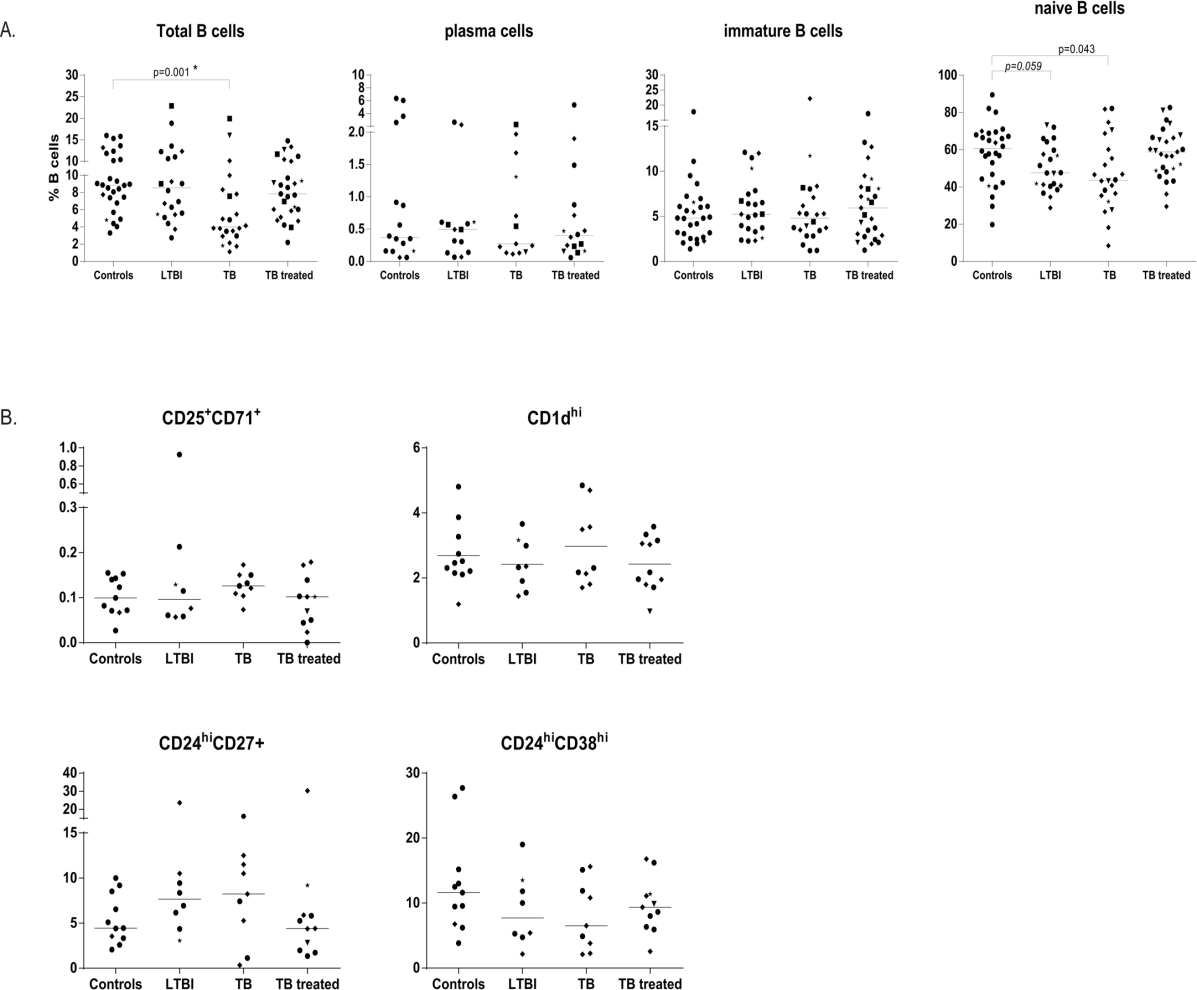
Patients with TB have less B-cells. PBMCs from individuals infected with Mtb (LTBI), with active TB disease or successfully treated TB patients and controls were thawed and stained directly for several combinations of B-cell surface markers. Lines indicate the median values of all groups. Ethnicity of donors was indicated using the following symbols: ‘black circle’ = West Europe; ‘black diamond’ = Est Europe; ‘black square’ = Africa; ‘black triangle’ = Asia; ‘black star’ = Sud America. Groups were compared to the uninfected controls using the Mann-Whitney test and a p< 0.05 was considered significant. * marks differences that remained significant after multiple test correction using Kruskal-Wallis testing with Dunn’s post-test. A. The percentage of CD19^+^ total B-cells in the different patients group expressed within the total lymphocyte population; CD20^-^CD21^-^ plasma cells expressed as percentage of CD19^+^ B-cells; CD10^+^ immature B-cells as percentage of CD19^+^ B-cells and IgD^+^CD27^-^ naïve B-cells as percentage of total CD19^+^ B-cells. The analysis was performed in 28 controls, 22 LTBI individuals, 22 TB patients and 27 TB treated subjects, except for plasma cells which were enumerated in 16, 14, 16, 13 individuals respectively. B. Regulatory B-cell populations were enumerated within the total CD19^+^ B-cells for a subset of individuals (11 control, 8 LTBI, 9 TB, 11 TB treated) by surface staining of B-cells within PBMCs.

The frequency of CD19^+^ B-cells was significantly decreased within the total lymphocyte population in patients with active TB disease compared to healthy controls (Fig 2A). Individuals with LTBI or treated TB individuals had frequencies of circulating CD19^+^ B-cells similar to controls (Fig 2A). Frequencies of plasma cells and immature B-cells did not differ among the evaluated subjects (Fig 2A); however, the frequency of naïve B-cells was decreased in patients with active TB and trended to a decreased frequency in individuals with LTBI compared to controls (Fig 2A). The frequency of CD19^+^ B-cells did not correlate with the levels of IgG antibodies reactive with PPD, Ag85B or ESAT-6/CFP-10 within the same individuals.

To assess the quality and specific characteristics of the B-cells from TB patients we investigated the distribution of regulatory B-cells (Bregs) and memory B-cell subsets. In contrast to previous studies [25,26] we did not observe any differences in CD1d^HI^ Bregs or CD25^+^CD71^+^ Bregs between any of the groups (Fig 1B). We also assessed the alternative CD24^HI^CD27^+^ and CD24^HI^CD38^HI^ Breg subsets, as they have been reported in other infectious diseases [27], however there were no significant changes observed between (treated) TB patients, LTBI individuals or controls (Fig 2B). Taken together, we did not observe significant changes in four different Breg populations.

**Fig 3:**
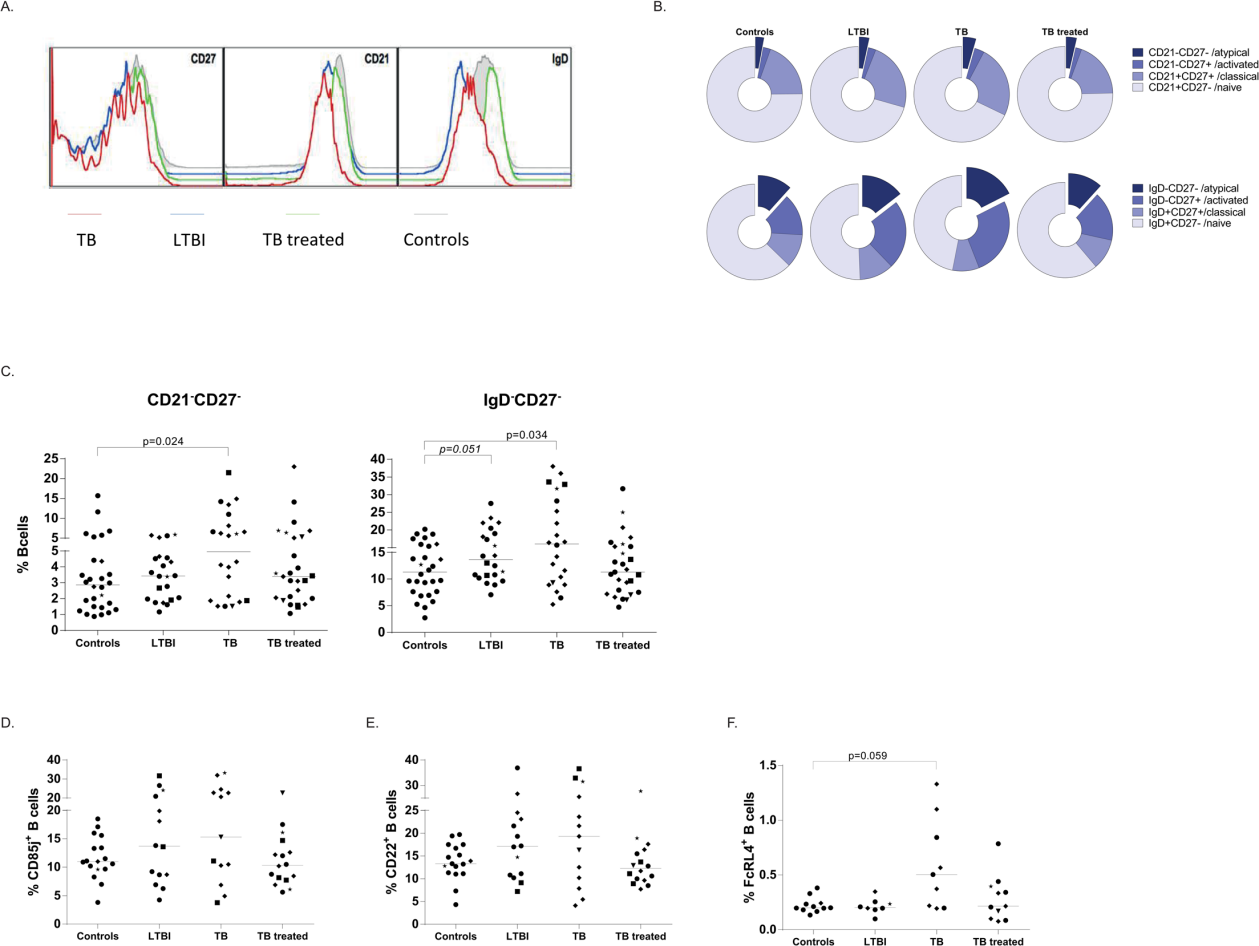
TB patients have an increased proportion of atypical B-cells. PBMCs were thawed and stained directly for several combinations of B-cell surface markers. Lines indicate the median values of all groups. Ethnicity of donors was indicated using the following symbols: ‘black circle’ = West Europe; ‘black diamond’ = Est Europe; ‘black square’ = Africa; ‘black triangle’ = Asia; ‘black star’ = Sud America. Mann-Whitney test was performed to compare infected groups to the uninfected controls and a p< 0.05 was considered significant. * marks differences that remained significant after multiple test correction using Kruskal-Wallis testing with Dunn’s post-test. A. Memory B cell subsets identified by concatenate analysis for CD27, CD21 and IgD on 17 control (grey), 14 LTBI (blue), 13 TB (red), 16 TB treated (green) individuals within the CD19^+^ B-cells. B. Memory B-cell subset distribution for CD21-CD27 (top row) and IgD-CD27 (bottom row) expressed as median of each group representing 28 controls, 22 LTBI individuals, 22 TB patients and 27 TB treated subjects. C. Atypical memory B-cells characterized by the absence of CD21 and CD27 (left panel) or the absence of IgD and CD27 for all individuals included in the study, expressed as percentage of total CD19^+^ B-cells, with a line at the median value. D. B-cell exhaustion markers CD85J (D), CD22 (E), FcRL4 (F) were measured on B-cells within total PBMCs and are expressed as percentage within the atypical memory B-cells.

B-cells can be divided in memory subsets based on the combined expression patterns of CD21 and CD27, or the combined expression of IgD and CD27. Naive B-cells are identified as CD21^+^CD27- or IgD^+^CD27-; classical B-cells as CD21^+^CD27^+^ or IgD^+^CD27^+^; atypical B-cells as CD21-CD27- or IgD-CD27- and activated B-cells as CD21-CD27^+^ or IgD-CD27^+^ (B-cell differentiation scheme in S4A Fig). Analysis of these three markers over the different groups, using concatenate analysis (which combines expression patterns of all individuals within a group) revealed an altered expression of CD21 and IgD in active TB patients and individuals with LTBI (Fig 3A; gating in S2 Fig). Analysis of the memory B-cell subsets during Mtb infection/disease showed an increased representation of double-negative atypical memory B-cells both in active TB patients and individuals with LTBI compared to controls and TB treated subjects (Fig 3B and 3C). In addition, active TB patients and individuals with LTBI had a larger proportion of IgD^-^CD27^+^ activated B-cells and fewer IgD^+^CD27^-^ naive B-cells (summary of data in S4B Fig).

Atypical, double negative, B-cells are also referred to as tissue-like memory B-cells or exhausted B-cells and have predominantly been described in chronic viral infections such as HIV [5]. They can be further characterized by the expression of exhaustion associated molecules such as FcRL4, CD85J and CD22. We have assessed the expression of these markers within the population of circulating atypical B-cells (IgD^-^CD27^-^), however we did not observe statistically significant differences between patents with active TB, LTBI, treated TB or controls (Fig 3D, 3E and 3F). Nevertheless, it was remarkable that some individuals with active TB disease or LTBI expressed these markers on a high proportion of their atypical B-cells. This suggests that both TB patients and LTBI subjects have an increased proportion of B-cells with an atypical phenotype.

### Lungs from deceased TB patients show aggregates of B-cells surrounding the granulomas

**Fig 4:**
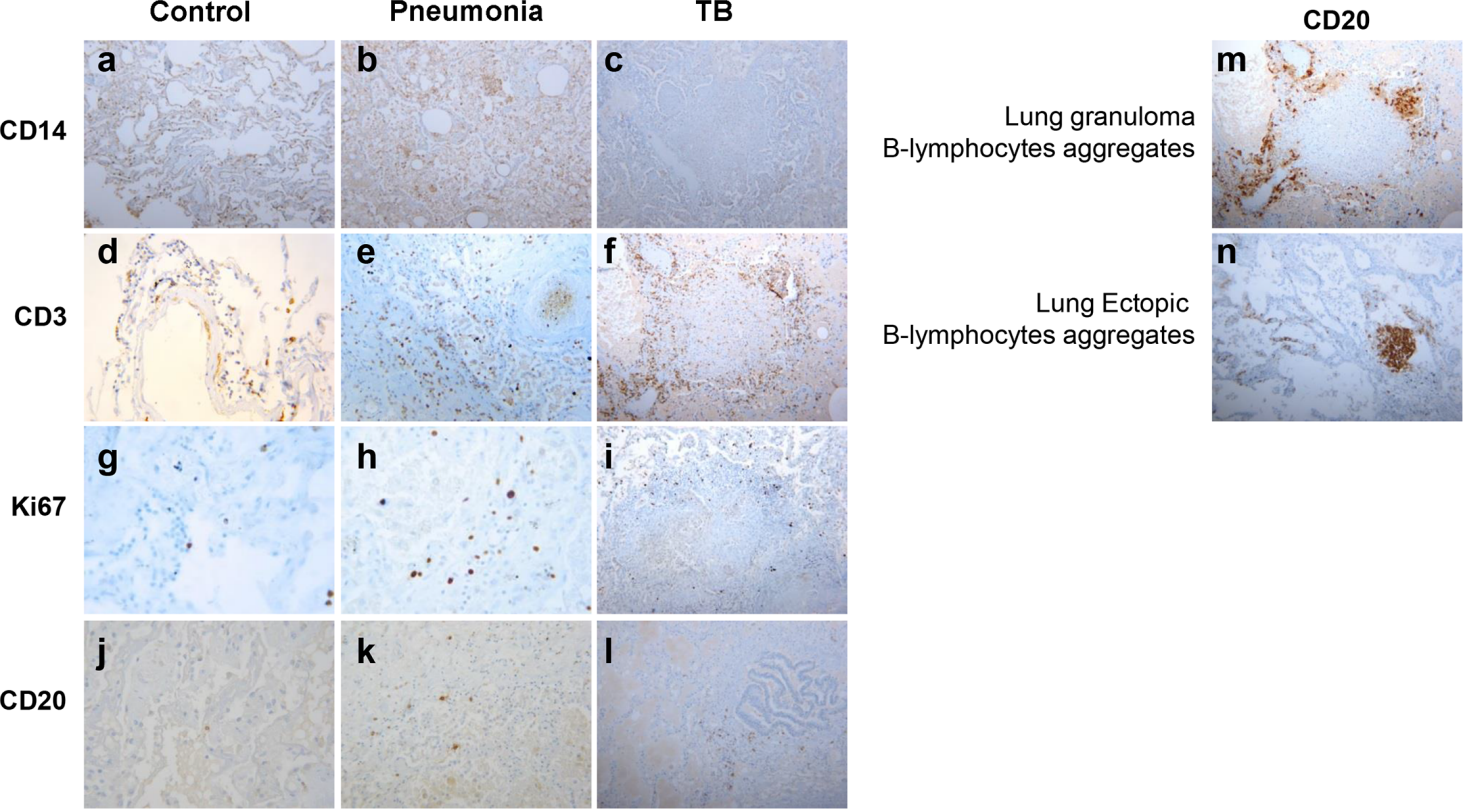
Lymphocyte, B cell and monocyte distribution in the lung. CD14, CD3 and CD20 cell distribution within lung tissues of patients died for TB, pneumonia, or causes other than pneumonia. Representative histological examination of lung specimens from autopsies of control patients died for causes other than pneumonia (n = 5) (a, d, g, j), for bacterial pneumonia (n = 10) (b, e, h, k) or for pulmonary TB (n = 10) (c, f, i, l). Samples were stained with CD14 Ab, CD3 Ab, CD20 Ab and Ki67 Ab. In TB patients B-cells are found mainly around the granuloma (m) or as aggregates near vessels (n). Original magnification (OM), 200x.

To assess the specific phenotypical and numerical characteristics of B-cells at the site of TB disease, an immunohistochemical analysis on the lung specimens from autopsies of patients with pulmonary TB was performed (Table 2). Samples from subjects with pneumonia different from TB or subjects who died from causes other than pneumonia, such as liver cirrhosis or heart failure, were evaluated as controls (Table 2). In particular, the samples from patients that had died from causes other than pneumonia displayed lung parenchyma characterized by fibrin aggregates, hyaline membrane or interstitial fibrosis as seen in respiratory distress syndrome. Therefore, only few inflammatory cells were found in these samples. The histological examination showed alveolar edema and interstitial inflammation consisting of CD14^+^ macrophages (Fig 4A) and CD3^+^ T-cells (Fig 4D). Rare CD20^+^ B-cells were observed (Fig 4L, 4M and 4N). Moreover, few Ki67 positive cells were found (Fig 4G). The lung specimens from subjects whom died from pneumonia not related to active TB were characterized by congested alveolar septa, airspaces filled with fibrin and neutrophils, with or without necrosis. Lymphocytes were only focally grouped or dispersed in the interstitial space. Pathological findings showed alveolar damage with neutrophilic infiltrates associated with abundant CD14^+^ macrophages (Fig 4B); CD3^+^ T-cells (Fig 4E) were found in the wall of vessel. Few Ki67^+^ cells (Fig 4H) and CD20^+^ B-cells were seen in the lung parenchyma (Fig 4L).

**Table 2:** Demographic and clinical information of the patients in whom an autopsy was performed.

		Controls	Pneumonia	TB
	N	5	10	10
**Age** median (IQR)		67 (60–76)	54 (37–75)	52 (37–65)
**Gender** Female, **N (%)**		3 (60)	4 (40)	3 (30)
**BCG, N (%)**				
	Vaccinated	-	3 (30)	6 (60)
	Unvaccinated	5 (100)	7(70)	4 (40)
**Origin, N (%)**				
	West Europe	5 (100)	7 (70)	4 (40)
	Est Europe	-	-	3 (30)
	Asia	-	1 (10)	-
	Africa	-	2 (20)	1 (10)
	Sud America	-	-	2 (20)
**Cavities, N (%)**				
	0	-		6 (60)
	1	-		4 (40)
	2	-		-
	>2	-		-
**Number of lungs involved, N (%)** *				
	1 lung	-		5 (50)
	2 lungs	-		5 (50)
**AFB in the smear, N (%)**				
	0	-		1 (10)
	1	-		-
	2	-		5 (50)
	3	-		4 (40)
**M. tuberculosis culture, N (%)**				
	positive			9 (90)
	negative			-
	ND			1 (10)
**M. tuberculosis molecular tests, (%)**				
	Positive			2 (20)
	Negative			-
	ND			8 (80)
**M. tuberculosis drug sensitivity to the first line drugs (%)**				
	sensitive			9 (90)
	resistant			-
	ND			1 (10)

TB: tuberculosis; AFB: Acid Fast Bacilli; LTBI: latent TB infection; BCG: Bacillus Calmette et Guerin; IQR: Interquartile Range; ND: not done

* all lesions: cavities, nodular lesions, consolidation

The lung samples from active TB patients showed granulomas characterized by a central region of caseous necrosis surrounded by foamy and epithelioid macrophages, as well as Langhans giant cells. Granulomas contained mainly CD14^+^ macrophages and CD3^+^ T-cells (Fig 4C and 4F respectively); a low Ki67 expression was found (Fig 4I). B cells were rarely found in the lung parenchyma (Fig 4L), as in patients with pneumonia (Fig 4K), and in the patients died from pneumonia other than TB; differently they were found mainly around the granuloma (Fig 4M) or in the outermost layer of the granuloma, near the vessels(Fig 4N), organized in pseudofollicles (Fig 4N) with a submembranous and membranous staining pattern. Based on the score used to classify the presence of CD20^+^ cells, 80% of the samples analyzed from the patients with active TB had a scoring higher than 1 (Table 3) whereas only 40% of the controls showed a score higher than 1 (Table 3); this difference was not significant. Representative images of CD20^+^ scores are shown in S3A–S3D Fig.

**Table 3:** Scoring of CD20 positive cells.

		Active TB	Pneumonia
N (SCORE)	% of positive cells evaluated in 10 fields per sample	N (%)	
1	0–10	2 (20)	6 (60)
2	10–25	4 (40)	3 (30)
3	25–50	3 (30)	1 (10)
4	50–75	1 (10)	0
5	>75	0	0

### B-cells from active TB patients and individuals with LTBI are functionally impaired

**Fig 5:**
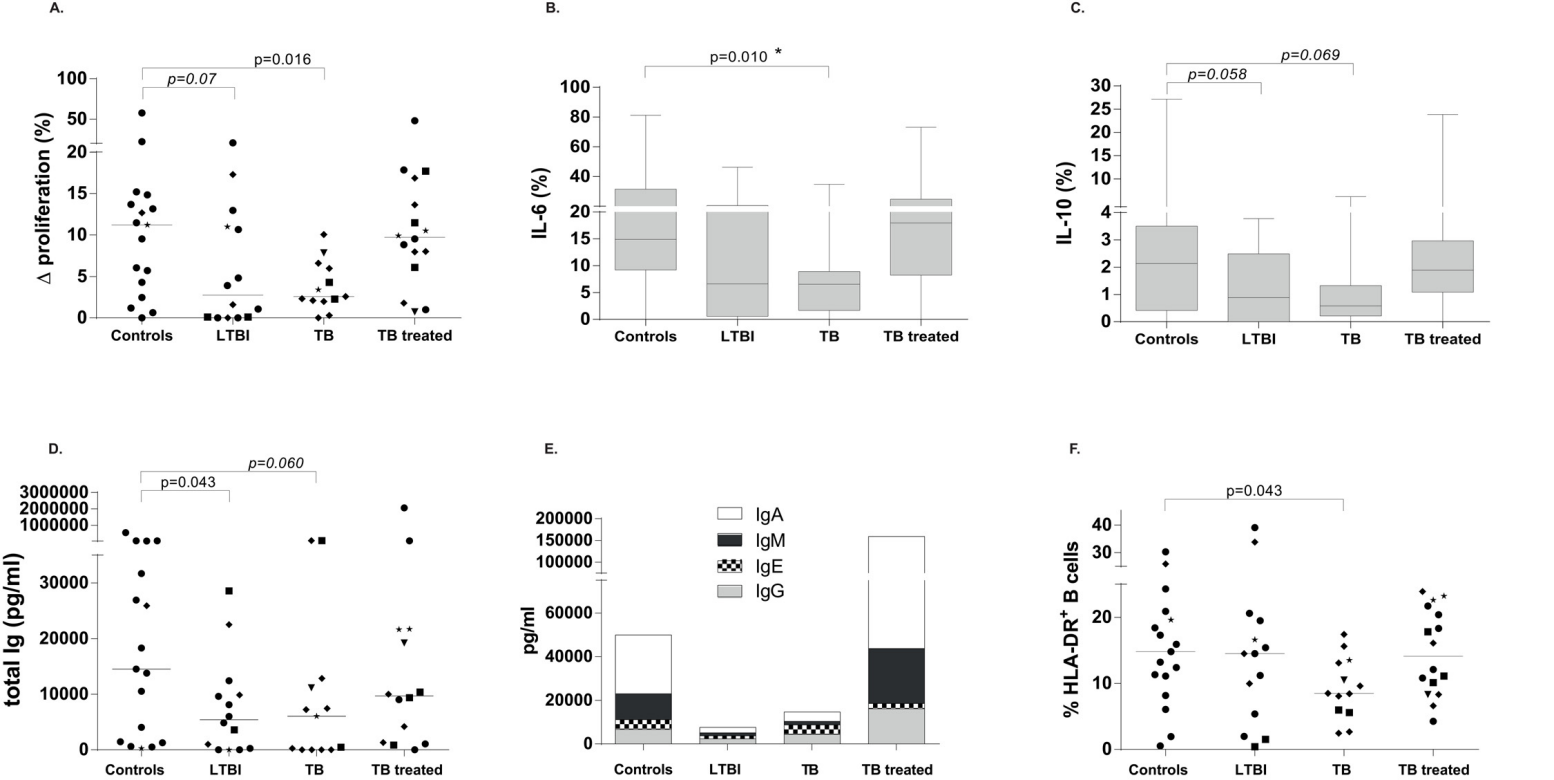
B-cells from TB patients as well as LTBI individuals have an impaired function. Total B-cells were isolated from PBMCs using magnetic bead separation for CD19 and stimulated with the combination of anti-CD40 and anti IgG/ IgM activating antibodies to obtain maximal, polyclonal B-cell activation. Data on isolated B-cells were obtained from 17 controls, 14 LTBI individuals, 13 TB patients and 16 treated TB patients. Lines indicate the median values of all groups. Ethnicity of donors was indicated using the following symbols: ‘black circle’ = West Europe; ‘black diamond’ = Est Europe; ‘black square’ = Africa; ‘black triangle’ = Asia; ‘black star’ = Sud America. Mann-Whitney test was performed to compare infected groups to the uninfected controls and a p< 0.05 was considered significant. * marks differences that remained significant after multiple test correction using Kruskal-Wallis testing with Dunn’s post-test. A. Isolated total CD19^+^ B-cells were labelled with a violet cell proliferation dye, proliferation in unstimulated conditions was subtracted from the anti-CD40 + purified IgG/ IgM stimulated conditions to obtain the delta proliferation reflecting the stimulation induced proliferation. Proliferation was assessed after 6 days of stimulation. B. Cytokine production measured by intracellular cytokine staining by flow cytometry for IL-6 (B) and IL-10 (C). Boxes express the 25–75% of data, with a line at median and whiskers indicate 5–95% data points. Cytokine production was measured after 2 days of stimulation; cytokines in unstimulated conditions were subtracted from stimulated conditions. C. Immunoglobulin production was measured in supernatants of unstimulated, isolated B-cells on day 2 of culture using multiplex bead array and expressed as total pg/ml for all isotypes combined. Lines indicate median values for each group. D. Immunoglobulin production per isotype, expressed as mean production per group of individuals for IgA, IgM, IgE and IgG. E. HLA-DR expression on B-cells following stimulation with anti-CD40 + anti IgG/ IgM expressed within the total CD19^+^ B-cells. Lines indicate median values.

Prompted by the finding of phenotypically atypical (CD21^-^CD27^-^ or IgD^-^CD27^-^) B-cells in the circulation of active TB patients, we isolated CD19^+^ B-cells from all groups and stimulated equal numbers of these cells with a strong polyclonal activation signal, consisting of anti-CD40 combined with anti-IgG/IgM antibodies to assess the functional properties of these B-cells. Proliferation, as measured using a cell cycle tracking dye, was strongly decreased in B-cells from both TB patients and showed a similar trend in B-cells from individuals with LTBI compared to healthy controls and patients after TB treatment (Fig 5A), implying a functional deficit in the B-cells of patients with active TB or LTBI. In addition to proliferation, also intracellular IL-6 was found to be decreased in B-cells from active TB and similar patterns observed in LTBI (Fig 5B). Intracellular IL-10 showed a similar trend (Fig 5C). Finally, as the hallmark of classical B-cell function, immunoglobulin (Ig) production was assessed. A reduced total Ig production by B-cells from LTBI subjects compared to controls was found (Fig 5D). Analysis of antibody isotypes revealed the strongest decrease in IgA and IgM levels compared to IgE or IgG (Fig 5E). Moreover, the B-cells from active TB patients expressed less HLA-DR, an activation marker for B-cells, following stimulation, compared to B-cells from the other groups (Fig 5F). Together these and the above results indicate that circulating B-cells from patients with active TB disease and LTBI -but not from treated TB patients- have an atypical phenotype and are functionally impaired.

### High T-cell activation in individuals successfully treated for pulmonary TB

**Fig 6:**
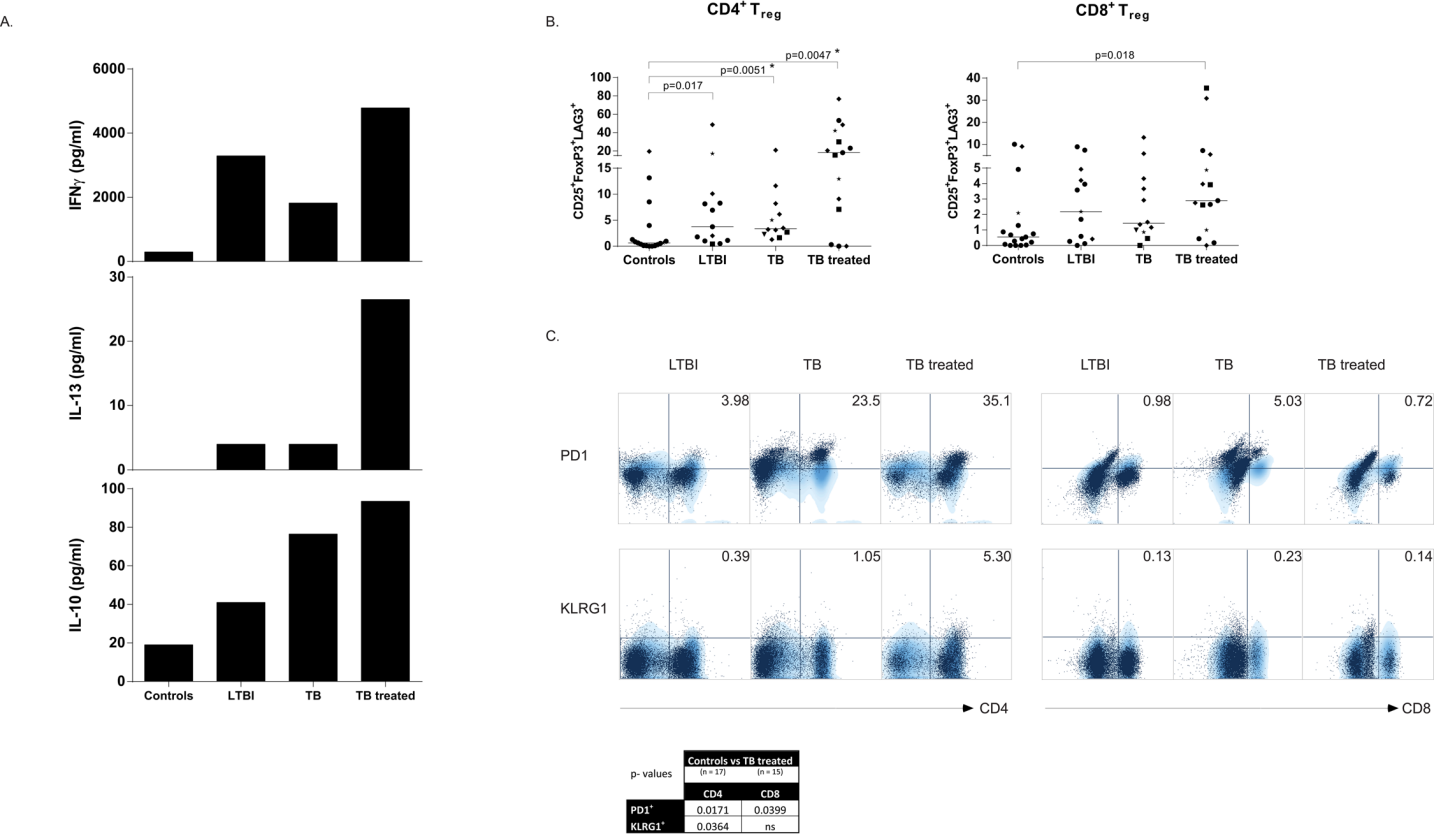
T-cell responses against mycobacteria are strongest in previously treated TB patients. PBMCs were stimulated with live BCG (MOI 3) for 6 days and CD4^+^ and CD8^+^ T-cells were analysed. The experiments were performed using cells from 17 controls, 13 LTBI individuals, 12 TB patients and 16 treated TB patients. Ethnicity of donors was indicated using the following symbols: ‘black circle’ = West Europe; ‘black diamond’ = Est Europe; ‘black square’ = Africa; ‘black triangle’ = Asia; ‘black star’ = Sud America. LTBI, TB and individuals treated for TB were compared to the controls using the Mann-Whitney test and a p < 0.05 was considered significant. *: p < 0.05; **: p < 0.01; ***: p < 0.001. * marks differences that remained significant after multiple test correction using Kruskal-Wallis testing with Dunn’s post-test. A. Cytokine production was measured in supernatants collected on day 5 by multiplex bead array and are expressed as median pg/ml in BCG stimulated minus unstimulated wells. B. Regulatory T-cell markers were stained by cell surface followed by intracellular staining, cells were gated on singlets, lymphogate, viable CD3^+^ T-cells followed by CD4 or CD8 gating and analysis of Treg associated markers using Boolean gating. Data are expressed as the percentage of CD25^+^FoxP3^+^LAG3^+^ cells within the CD4^+^ (left) or CD8^+^ (right) gate of BCG stimulated minus unstimulated samples, with a line at median. C. Inhibitory marker expression on CD4^+^ (top) and CD8^+^ (bottom) T-cells following BCG stimulation, light blue represents a healthy control with an overlay in dark blue from a Mtb infected individual. Mann-Whitney analysis on all individuals compared to the control group revealed a significant increase of PD1 on CD4^+^ (p = 0.017) and CD8^+^ (p = 0.039) T-cells of TB treated individuals. KLRG1 expression was only significant on CD4^+^ T-cells (p = 0.036) of TB treated subjects.

As T- and B-cells closely interact during the adaptive immune response we next investigated T-cell activation across the respective stages of TB infection/ disease. To this end, PBMCs were stimulated with live *M*. *bovis* BCG for 6 days and cytokine production as well as CD4^+^ and CD8^+^ T-cell subset involvement were assessed. We have previously shown that 6 day live BCG stimulation results in activation of mycobacterium-specific T-cells with regulatory capacity (Treg) and therefore assessed Treg frequencies here as well [28,29]. Secretion of Th1 (IFNγ) or Th2 (IL-13) cytokines in supernatants of BCG stimulated PBMCs was observed in all groups infected with Mtb, with the highest levels detected in individuals successfully treated for TB (Fig 6A). Secretion of IL-10 was also observed in all groups, albeit at low levels, with the highest levels again in treated TB patients.

Analysis of T-cell phenotypes focussed on the identification of regulatory T-cells (Tregs) and T-cells expressing inhibitory receptors associated with exhaustion, in order to assess the possible association between ‘exhausted’ atypical B-cells and exhausted T-cells (S4 Fig). CD4^+^ and CD8^+^ Treg marker expression, defined as CD4 or 8, CD25, FoxP3 and LAG3, were assessed; CD4^+^ Tregs were induced in all TB groups whereas CD8^+^ Tregs were significantly increased in TB treated patients (Fig 6B). Intriguingly, the population of CD4^+^ Tregs was most abundantly detected, and at a very high frequency, in patients previously treated for TB. Similarly, but less dramatically different, also the proportion of CD8^+^ Tregs was highest in previously treated TB patients, possibly as a result of the Mtb antigenic load which is released during following TB chemotherapy.

T-cell exhaustion is typically defined by the expression of the inhibitory receptors PD1 and/or KLRG1. We mostly observed PD1 expression on CD4^+^ T-cells across the different Mtb-exposed groups, predominantly in TB treated subjects. Among CD8^+^ T-cells, we observed PD1 expression in active TB. In contrast, KLRG1 expression was significantly detected on CD4^+^ T-cells of TB treated subjects and was not increased in LTBI individuals or TB patients compared to controls, whereas CD8^+^ T-cells showed no differential expression of KLRG1 among the analysed groups (Fig 6C) [30]. These data thus strongly suggest that T-cell exhaustion and B-cell exhaustion are independently regulated and do not necessarily occur in the same individuals at the same time.

**Fig 7:**
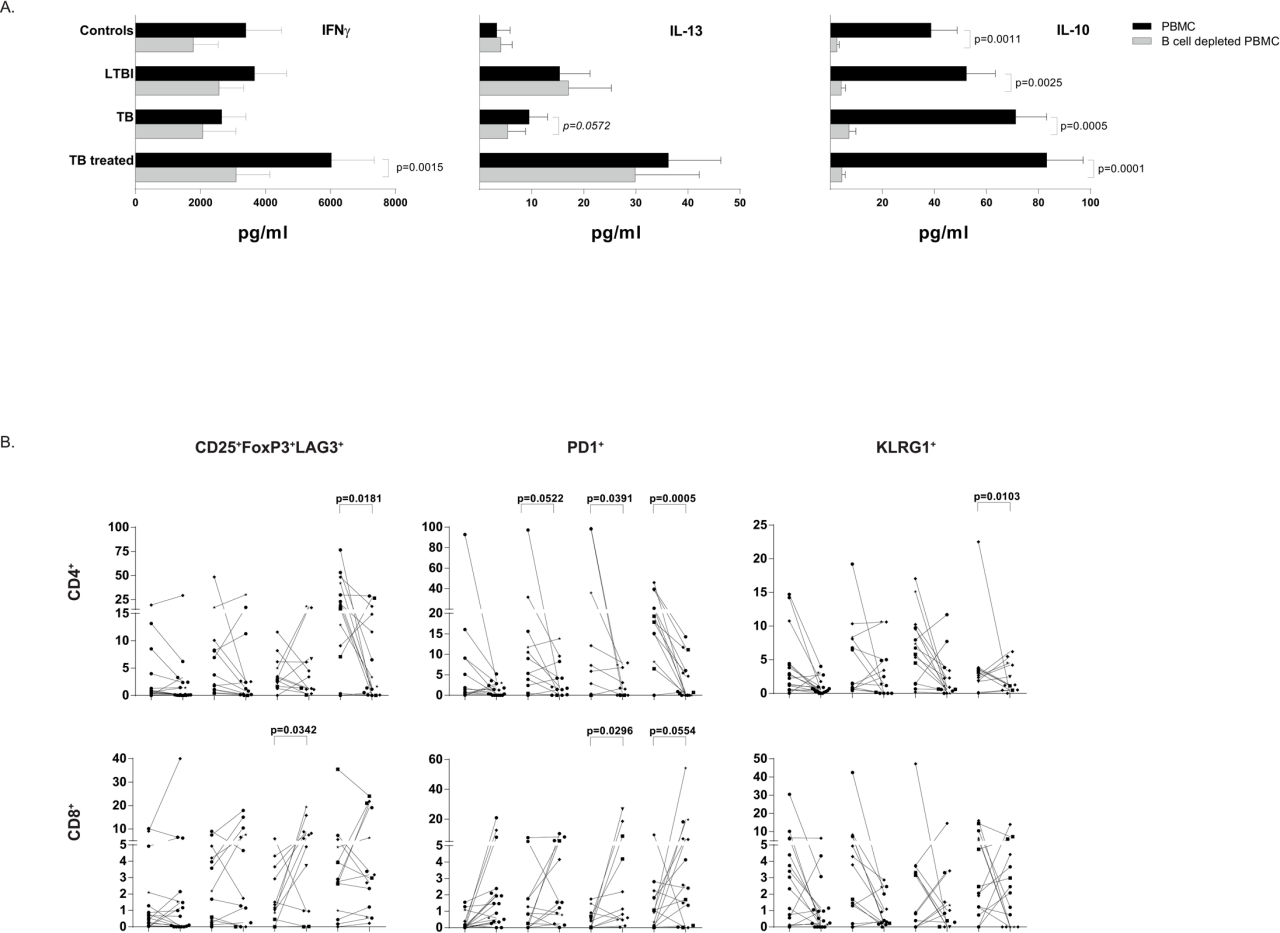
B-cells contribute to the magnitude of T-cell responses in treated TB patients. To investigate the contribution of B-cells to T-cell activation in the various groups of Mtb infected individuals we depleted CD19^+^ B-cells from PBMCs and compared the T-cell responses following BCG stimulation in total PBMC to those in B-cell depleted PBMCs. Ethnicity of donors was indicated using the following symbols: ‘black circle’ = West Europe; ‘black diamond’ = Est Europe; ‘black square’ = Africa; ‘black triangle’ = Asia; ‘black star’ = Sud America. Data obtained from B-cell depleted samples were compared pair-wise to total PBMC samples using the Wilcoxon signed Rank test and a p-value < 0.05 was considered significant. *: p < 0.05; **: p < 0.01; ***: p < 0.001; @: p = 0.052 (PD1 on CD4^+^ T-cells in LTBI), p = 0.055 (PD1 on CD8^+^ T-cells in TB treated patients s). A. Cytokine production (median in pg/ml) in supernatants on day 5 following BCG stimulation (medium subtracted) for total PBMC (black) and B-cell depleted PBMC (grey). Data are expressed as mean + standard error of the mean. B. Expression of regulatory T-cell markers, or exhaustion markers PD1 and KLRG1 (as percentage of CD4^+^ or CD8^+^ T-cells) in total PBMCs (filled dots) or following CD19 depletion (open dots).

As B-cells are central players in the adaptive immune response, B-cell malfunction may contribute to the decreased T-cell responsiveness observed in subjects with LTBI or active TB compared to those successfully treated for TB. Therefore, T-cell responses were analysed in total PBMCs and compared to B-cell depleted PBMCs. Depletion of B-cells had the strongest effect on T-cell activation in patients previously treated for TB, whom had fully functional B-cells as shown above. In contrast, B-cell depletion had only a minor effect on cytokine responses in active TB patients and individuals with LTBI, both of whom had dysfunctional B-cells. Both Th1 and Th2 cytokine responses strongly decreased following B-cell depletion of PBMCs in treated TB patients (Fig 7A). IL-10 production in supernatants was almost completely abrogated following B-cell depletion in all samples (Fig 7A). Interestingly, B-cell depletion resulted in a significant decrease in the frequency of CD4^+^ Tregs compared to total PBMCs in treated TB subjects, but not in active TB patients or LTBI individuals (Fig 7B). These data indicate that depletion of B-cells in TB patients does not affect T-cell activation, supporting again the poor functionality of B-cells during active Mtb replication. CD8^+^ Tregs increased following B-cell depletion in patients with active TB (Fig 7B). Similarly, PD1 expressing CD4^+^ T-cells and to a lesser extent also KLRG1 expressing CD4^+^ T-cells decreased following B-cell depletion, to the largest extent in treated TB patients (Fig 7B). Intriguingly, as also seen for CD8^+^ Tregs, CD8^+^ T-cells expressing PD1 increased significantly.

Thus depletion of functional B-cells such as in treated TB subjects diminished BCG induced cytokine production and T-cell activation, possibly as a result of APC depletion. In contrast, depletion of functionally impaired B-cells in LTBI individuals or TB patients hardly affected T-cell responses, which implies that functional B-cells are a critical component determining the magnitude of the antimycobacterial-specific Th1 and Th2-cell response.

## Discussion

The functional significance of B-cells in the control of Mtb infection in humans has been under studied compared to the large number of studies assessing the contribution of T-cells. Literature on B-cells in TB has suggested that these cells, apart from the antibodies they produce, may be involved in disease pathogenesis but data are scarce and often contradictory. We therefore decided to analyse phenotype and function of B-cells during Mtb infection and disease; active pulmonary TB, successfully treated TB, recent LTBI and control uninfected individuals. We specifically analysed B-cell memory- and exhaustion-phenotypes, B-cell functions (proliferation, cytokine and immunoglobulin production) as well as their ability to induce T-cell activation. We demonstrate not only that circulating B-cell numbers are decreased in patients with active TB disease, but more importantly—for the first time to the best of our knowledge- that B-cells from individuals with active disease or recent LTBI are functionally impaired. We identified functionally impaired B-cells in (recent) LTBI and active TB patients, but not in treated TB subjects. B-cells thus seem impaired occurs during active Mtb replication, which likely happens also in recent LTBI, however the mechanism responsible for this remains unknown. Alternatively or additionally, inflammatory processes may be involved in down regulation of B-cell functions. Moreover, B-cells from LTBI and active TB patients had also impaired functional capacities following polyclonal activation, including impaired cell proliferation, impaired cytokine production as well as impaired immunoglobulin production. These impairments were more prominent in patients with active TB compared to LTBI individuals, and varied for the different parameters. In particular, individuals with LTBI showed considerable variation in their responses which may reflect the heterogenic “spectral” nature of this group, ranging from active infection to presumably sterile eradication of Mtb infection. The variation in responses observed in all groups is considerable, reflecting the variation in TB disease phenotype or even inter-individual variation.

Our data thus highlight abnormal B-cell numbers and memory B-cell distribution in patients with active TB as well as a functional impairment of B-cells during active Mtb replication. Moreover, we show that B-cells are functionally involved in T-cell activation, including activation of regulatory T-cells, because depletion of B-cells significantly affected these cell populations particularly in treated TB patients.

In TB disease, the role of immunoglobulins, the primary products of B-cells, is under debate: although antigen-specific immunoglobulins are highly produced by TB patients their contribution to bacterial and/or infection control is less clear [31]. Antibodies from patients with active TB disease have been reported to recognize a different repertoire of Mtb antigens than those from individuals with controlled latent TB, and antibody titers correlated with mycobacterial burden [32]. Here, we also detected antibodies against mycobacterial PPD in patients with active and treated TB disease but not in those with LTBI, antibodies against Ag85B were detected in all infected groups albeit at very low levels. Interestingly, antibody levels did not correlate with total B-cell frequencies within the same sample, patients with very low B-cell frequencies still had average levels of PPD reactive antibodies. Importantly, we did not observe differences in the antibody producing plasma cell frequencies between any of our groups (Fig 2A). Plasma cells differentiate from activated memory B-cells (S5A Fig), a population that is relatively upregulated in LTBI and patients with active TB, suggesting that Mtb does not alter the normal development of antibody producing cells and thus also allow the production of Mtb specific antibodies. We did not observe differences in antibody levels between patients with active TB disease and those successfully treated for TB, supporting that plasma cell differentiation and function of differentiated plasma cells is not affected in a similar matter as total B-cell function in those with active TB disease.

The impact of antibodies in TB remains debated [32],for a long time they have been considered inefficacious because Mtb resides within the macrophages. However, more recently it has been demonstrated that immunoglobulins can contribute to the elimination of intracellular pathogens and thereby reduce tissue damage (reviewed in [7,32]). Potential mechanisms that have been implicated include opsonisation, enhanced phagolysosomal fusion mediated through FcγR signalling, and increased intracellular killing of Mtb following Ca2+ flux [32]. A recent paper demonstrated that IgG antibodies directed against the Mtb capsular polysaccharide arabinomannan contributed to enhanced phagocytosis and inhibition of intracellular Mtb growth [33]. Moreover, it has been shown that antibody binding to pathogens may also be detected intracellularly via a cytosolic Fc-receptor called TRIM21 [34,35]. Pathogen bound antibody triggering of TRIM21 resulted in immune activation and inflammatory signals [34]. TRIM21 expression was increased in the blood of active TB patients compared to healthy controls, and expression patterns of TRIM21 grouped with those of other Fc receptors [22,23].

The decreased frequencies of B-cells in the circulation of TB patients could have been the result of B-cell homing to the site of disease. Our immunohistochemical analyses showed an increased presence of B-cells in lung tissues from TB patients compared to the lungs of patients that died from causes different from pneumonia. These B-cells were associated to the periphery of the granuloma and to ectopic follicles close to the vessels as previously described and explained as cells needed to orchestrate the immune response against Mtb outside the granuloma [20]. However this B-cell increase in the lung appeared modest compared to the B-cell depletion observed in the circulation which appeared severe. Unfortunately, we were not able to analyse paired tissue and peripheral blood samples from the same patients to investigate a causal relation. We do appreciate that those unfortunate patients that succumbed from the active TB may not reflect the normal disease process as they represent the most severe end of the disease spectrum. Yet, analysis of this tissue material has provided us insight in the B-cell pattern during severe TB disease locally in the lung. In addition to their decreased frequencies, also B cell function is severely hampered in patients with active TB disease, with a similar trend in LTBI individuals. Patients that have successfully completed the 6 month treatment regimen for pulmonary TB (studied time points ranged from 1–72 months post treatment completion) have fully restored B-cell numbers and functions, comparable with healthy uninfected controls. This suggests that chemotherapeutic elimination or reduction of Mtb replication and metabolism, and consequent reductions in inflammation are related to the functional restoration of the B-cell compartment.

Moreover, our data suggest that B-cells with phenotypical characteristics of exhaustion are not necessarily exhausted, but can recover, or at least repopulate, the circulation with normally functioning B-cells. This is in line with what has been reported previously for HIV-infected patients in which treatment with anti-retroviral therapy also resulted in abrogation of FcRL4 expressing atypical B-cells [5,36], as well as for individuals infected with Schistosomes in which the frequency of atypical B-cells was increased during infection and decreased to normal levels following treatment [37]. The B-cell unresponsiveness that we observe here may not only impact immunity towards Mtb itself but also other unrelated antigens and pathogens, as we find a strong and generalized impairment in the functional properties of B-cells following polyclonal stimulation. Individuals with LTBI in our study were recent contacts from active TB patients, although their previous mycobacterial exposure is unknown. It may well be that LTBI individuals exposed and infected more remotely in time are less affected and have normo-functional B-cells. There are no previous reports as far as we know of LTBI having poor antibody responses following vaccination or infection with other pathogens. This would agree well with our hypothesis that B-cell impairment by Mtb is transient and associated with active bacterial replication such as early following infection and during TB disease or with active ongoing inflammation. However, due to the cross-sectional design of our study we cannot exclude that intrinsic defects in B-cells are responsible for the development of TB disease in patients, nor can we formally prove that the B-cell defect is acquired as a result of Mtb infection.

Individuals treated for TB disease had the highest T cell cytokine production as well as the highest frequency of T-cells expressing Treg markers, indicating a high level of T-cell activation in these subjects. The majority of TB treated patients analysed in this study had completed their treatment relatively recently such that memory T-cells should still be abundantly present after the likely massive Mtb antigen release induced by antibiotic treatment. However, only T-cell responses in treated TB patients diminished following B-cell depletion, suggesting that only normally functioning but not atypical-/exhausted B-cells contributed to the augmentation of T-cell activity. In support of this notion, depletion of B-cells which appeared largely exhausted from samples of TB patients or individuals with LTBI indeed did not augment (nor diminish) T-cell responses to BCG stimulation.

*In vivo* in mice, B-cell deficiency did not result in hampered T-cell cytokine responses [14,15] however, the protracted time scale of the *in vivo* measurements is completely different from the *in vitro* analyses performed here. Recently, B-cells were depleted in an acute Mtb infection model in Rhesus Macaques and although the pathology and clinical outcome were not different, local analysis in the tissue, and in particular in the granuloma’s revealed differences in local T-cell responses and cytokine production [18]. Variation between individual granulomas was observed within the same animals, but there was a clear indication that B-cell depletion affected local inflammation. In addition, *in vivo* tissue resident professional APCs such as dendritic cells and macrophages may compensate for the deficiency of B-cells as APCs in those models.

To the best of our knowledge, this is the first report on exhausted B-cells induced by a bacterial infection. Intriguingly and in contrast to our data described here, children chronically exposed to a parasite as the *Plasmodium falciparum*, had both atypical B-cells and exhausted T-cells [38]. In our study reported here, exhausted T-cells were most predominant in treated TB patients, whereas atypical B-cells were most prominent during active disease, suggesting that their induction is independently regulated. Perhaps systemic antigen exposure is already increased during blood stage malaria disease, with continued cycles of parasite release, as compared to TB, where massive antigen release occurs mostly after initiation of treatment, and therefore differences in kinetics may occur.

A possible limitation of the data we present here is the use of cross-sectional groups of TB patients and treated TB patients. A longitudinal follow up of individual patients would be highly valuable to better understand the kinetics of B-cell and T-cell subset populations. Similarly, longitudinal follow up of individuals with recent LTBI will also be valuable to determine the duration of B cell impairment. Moreover, matching of in particular TB patients and controls was suboptimal in terms of sex and ethnicity.

In conclusion, we have shown here that B-cells from patients with active TB and with recent LTBI, are decreased in numbers, have a phenotype of atypical B cells and are functionally impaired. TB treatment restores B cells in terms of key phenotypic and functional features—Moreover in the group of TB-treated patients highest levels of T-cell activation, including cytokine production, and expression of Treg markers and inhibitory receptors was observed. Mtb specific antibodies can be detected to a similar extent in active TB and treated TB patients. Moreover, B-cells appear central in antigen specific T-cell activation since B-cell depletion dramatically abrogated the potent T-cell activation in treated individuals. Together these data demonstrate that B-cell function is impaired during active TB disease, and that this has important consequences for T-cell activation, which may affect the overall host immunity towards TB.

## Materials and Methods

### Experimental design

This is an experimental laboratory study performed with human peripheral blood samples. This study was designed to describe B-cell numbers, phenotype and function during Mtb infection and TB disease before and after specific treatment. Well-characterized patients were recruited at the National Institute of Infectious Diseases in Rome, Italy (Table 1). Patients received standard care and blood samples were collected for research purposes. The study was approved by the local ethical committee and all subjects provided informed consent. The number of individuals included is indicated in the tables and figure legends. All clinical samples were anonymized by laboratory codes and tested and analysed blinded to their clinical status in all assays. Once the experimental results were completely obtained the codes were revealed to know the corresponding clinical status.

### Ethics statement

This study was approved by the Ethical Committee of the L. Spallanzani National Institute of Infectious diseases (INMI), approval number 02/2007 and 72/2015. Informed written consent was required to participate in the study and was obtained before collecting blood samples.

Collection and use of histological samples was approved by the ethical committee of INMI, approval number 72/2015. Informed consent was not required for use of histology samples obtained at autopsy.

### Study subjects for cellular analysis

Active pulmonary TB was sputum culture-confirmed and patients were enrolled within 7 days of starting the specific treatment. Treated TB subjects were patients who had completed a 6-month course treatment for culture-positive pulmonary TB and who were culture-negative after 2 and 6 months of therapy. An additional group of patients was evaluated after therapy completion (1–72 months after end of therapy). LTBI was defined based on positive response to Quantiferon (QFT-IT) in a healthy subjects without radiological signs of active disease [39,40]. LTBI subjects were mainly contacts recently exposed (in the previous 6 months) to smear-positive active pulmonary TB patients (15/22), however infection may have occurred also at earlier time points as no information (QFT-IT or TST) is available on any prior time points. Healthy, uninfected controls were QFT-IT negative individuals. All enrolled subjects tested negative for HIV and were not undergoing treatment with immunosuppressive drugs. Demographic and epidemiological information were collected at enrolment (Table 1).

The control group was complemented with 10 anonymous, Dutch, healthy adult blood bank donors (Sanquin blood bank, Leiden) that tested negative for *in vitro* recognition of mycobacterial PPD (purified protein derivative). PPD-reactivity was tested by stimulation of PBMCs with 5 μg/ml PPD (Statens Serum Institute, Copenhagen, Denmark) for 6 days and supernatants were tested in an Interferon (IFN)γ-ELISA (U-CyTech, Utrecht, The Netherlands). PPD positive responses were defined as IFNγ-production > 150 pg/ml.

PBMCs were isolated by Ficoll density centrifugation and were stored and shipped in liquid nitrogen until use.

### Study subjects for immunohistochemical analysis

Collection and use of histological samples was approved by the ethical committee of INMI, approval number 72/2015. Tissues were obtained during the autopsies from 10 subjects with pulmonary tuberculosis (sputum or broncho-alveolar-lavage positive for Mtb-culture or PCR), 10 subjects with pneumonia other than active TB and 5 patients died from causes other than pneumonia, such as liver cirrhosis or heart failure. Demographic, clinical and pathological features of all cases were collected and are summarized in Table 2.

### Specific antibody ELISAs

Plasma samples from active and treated TB patients, latently infected individuals and controls were tested for the presence of Mtb specific antibodies. Flat bottom, 96-well Microlon plates (Greiner) were coated with 5 μg/ml PPD (Statens Serum Institute, Copenhagen, Denmark), 5 μg/ml Antigen 85B or 5 μg/ml ESAT-6/CFP-10 fusion protein [41] in PBS or 2 hours at 37°C. Plates were blocked with PBS/ 1% BSA/ 1% Tween 20 for one hour before incubation with 6 serial dilutions of plasma (1:25–1:800), diluted in PBS/ 1% BSA/ 0.05% Tween 20 and incubated at room temperature overnight. IgG antibody binding was detected using HRP labelled polyclonal rabbit anti-human IgG antibodies (Dako, Glostrup, Denmark) followed by TMB substrate buffer (Sigma Aldrich), stopping the color reaction using H_2_SO_4_ and OD450 reading. The EC50 was determined using a non-linear curve fit and was defined as the midpoint of the linear portion of the dilution curve.

### Flow cytometric analysis

For analysis of B-cell subsets PBMC were thawed and stained for 10 minutes at 4°C with the violet fixable live dead stain (Vivid, LifeTechnologies Europe-Invitrogen, Bleiswijk, Netherlands) according to the manufacturer’s protocol, immediately followed by a 30 minutes staining at 4°C for CD19 biotin (clone HIB19; Biolegend, ITK diagnostics, Uithoorn, The Netherlands) and Streptavidin Brilliant Violet510 (BD Biosciences, Erembodegem, Belgium), CD25 APC-H7 (clone M-A251;), CD27 APC (clone L128,), CD38 FITC (clone HIT2; all BD Biosciences) CD24 PE-Cy7 (clone ML5; Biolegend), CD71 PE-Cy5 (clone M-A712, BD Biosciences), and CD1d PE (clone 51.1; eBioscience, Vienna, Austria) for Breg analysis. Plasma cells and memory B-cells were enumerated by staining with CD27 FITC (clone M-T271, BD Biosciences), FCRL4 PE (clone 412D12, Biolegend), CD10 PerCP-eFluor710 (clone eBioSN5c, eBioscience), CD19 PE-Cy7 (clone HIB19, Biolegend), CD21 APC (clone B-ly4, BD Biosceinces), IgD biotin (clone AI6-2, BD Biosciences) with Streptavidin Qdot525 (LifeTechnologies Europe-Invitrogen) or CD10 FITC (clone HI10a), CD20 APC-H7(clone 2H7), CD27 APC (clone L128), IgD HorizonV500 (clone IA6-2), CD22 PE-Cy7(clone HIB22), HLA-DR PE-Cy5 (clone G46-6), CD21 PE-CF594 (clone: B-ly4), CD85j PE (clone GHI/75) (all BD Biosciences) and FCRL4 PerCP-eFluor710 (clone 413D12) in the presence of purified human FcR binding inhibitor CD16/CD32 (both eBioscience). After staining cells were washed with PBS/BSA 0.1% (Roche, Woerden, The Netherlands) and fixed with 1% paraformaldehyde (LUMC pharmacy, the Netherlands) prior to analysis.

B-cell proliferation was further assessed by flow cytometry. After a total of 6 days of culture, B-cells were harvested and the surface was stained with CD20 APC-H7, CD22 PE-Cy7, HLA-DR PE-Cy5, CD21 PE-CF594, CD85j PE (all BD Biosciences) and FCRL4 PerCP-efluor710 in the presence of purified human FcR binding inhibitor (eBioscience). Cells were washed, fixed for 15 minutes using the Fixation buffer A (ADG, ITK Diagnostics, Uithoorn, The Netherlands), washed once more and stained for intracellular IL-6 (clone: MQ-13A5, BD Biosciences) and IL-10 (clone: JES3-9D7, Miltenyi Biotec BV, Leiden, The Netherlands) in Permeablization buffer B (ADG) for 30 minutes at RT. Cells were washed for the last time and immediately acquired on a BD LSRFortessa.

T-cells were analyzed for T-regulatory markers and for inhibitory receptor markers using the following fluorochrome panel after staining with the violet fixable live dead stain (Vivid, Life technologies); for surface staining, CD3 Brilliant Violet570 (clone UCHT1), KLRG1-Pe-Cy7 (clone 2F1/KLRG1)(both Biolegend), CD4 PE-CF594 (clone S3.5; LifeTechnologies Europe-Invitrogen), CD8 HorizonV500 (clone RPA-T8), PD-1 PerCP-Cy5.5 (clone EH12.1)(both BD Biosciences) were used. After 30 minutes cells were washed with PBS/BSA 0.1%, fixed with fixation buffer A for 15 minutes at RT, washed and stained for FoxP3-FITC (clone PCH101; eBioscience), CD25 APC-H7 (clone M-A251; BD Biosciences) and LAG3-Atto647 (clone 17B4; Enzo Life Sciences BVBA, Raamsdonksveer, the Netherlands), in permeabilization buffer B for an additional 30 minutes at RT. Finally, cells were washed once more and analyzed.

All acquisitions were done on a BD FACS Canto or a BD LSRFortessa with FACS Diva software version 6.2. Analysis was performed with FlowJo software version 9.7.6. Gating strategies are provided in compliance with the MIATA guidelines in S2 and S3 Figs [42].

### Isolation and stimulation of B-cells

PBMCs were incubated with CD19 microbeads according to manufacturer’s protocol (Milteny Biotec BV, Leiden, The Netherlands) to isolate B-cells. After positive selection cells were checked for purity and in 76% of the cases the cells were enriched for more than 90%, while in the remaining 24%, the B-cells were between 80–90% purity. The CD19 depleted fraction was further used for the analysis of Bacillus Calmette Guerin (BCG) stimulated T-cell responses in the absence of B-cells and compared to total PBMC responses. B-cells were stained with 1μl per 10x10^6^ cells/ml of the CellTrace violet cell proliferation kit (Invitrogen) and incubated for 20 minutes at room temperature. Reaction was stopped by adding Fetal Bovine Serum (FBS) rich medium according to manufacturer’s protocol. After labeling, the B-cells were plated in 96 wells round bottom plate at 3x10^5^ cells/well and cultured in RPMI supplemented with 100 U/ml penicillin, 100 μg/ml streptomycin, 1mM pyruvaat and 2mM glutamate (all Gibco Life technologies, Thermo Fisher Scientific Inc, Merelbeke, Belgium) + 10% FBS (Greiner Bio-one, Alphen a/d Rijn, The Netherlands) with medium only or a polyclonal stimulation of αCD40+ anti IgG/M (αCD40 at 1 μg/ml, Biolegend, ITK Diagnostics, Uithoorn, The Netherlands; 10 μg/ml AffiniPureF(ab’)2 Fragment Goat Anti-Human IgG + IgM (H+L), Jackson ImmunoResearch Laboratories inc., Suffolk, UK) for 5 days. For the last 18 hours Brefeldin A (3 μg/ml, Sigma-Aldrich Chemie BV, Zwijndrecht, the Netherlands) and Monensin (1/1000, Biolegend) were added. Supernatants of these cultures were harvested at day 2 and day 5 for subsequent determination of immunoglobulin levels.

### BCG preparation

The BCG (Pasteur strain) was grown in Middlebrook 7H9 medium supplemented with 10% ADC (BD Biosciences), log phase bacteria were used for infection experiments. Multiplicity of infection was calculated after determination of the number of viable bacilli per ml inoculum by plating serial dilutions of bacteria on Middlebrook 7H10 agar plates supplemented with 10% OADC (BD Biosciences) and counting of visible colonies after 3 weeks. Infections of PBMCs were done at a multiplicity of infection (MOI) of 3.

### Stimulation of PBMC and CD19-depleted PBMC with BCG

PBMCs (1x10^6^ cells/well) or the CD19-depleted PBMC fraction (1x10^6^ cells/well) were stimulated in a 48 wells plate with or without live BCG at a MOI of 3 for 6 days as previously described, resulting in amongst others functional Treg activation [29]. T-cells were cultured in IMDM supplemented with glutamax (Gibco Life Technologies) but without antibiotics. Supernatants were collected at day 2 and day 5 for cytokine analysis and cells were harvested at day 5 for flowcytometric analysis.

### Luminex

Immunoglobulins were tested in an 1 to 5 dilution using the ProcartaPlex for human Isotyping (Affymetrix- eBioscience, Vienna, Austria) according to manufacturer’s instructions.

T-cell supernatants were measured with a Bio-plex luminex kit for human IL-10, IL-13 and IFN-γ (Bio-rad, Veenendaal, The Netherlands). Luminex was performed according to manufacturer’s instructions and analyzed on a Bio-Plex 200 system with Bio-plex software (Bio-rad).

### Immunohistochemical staining and quantification

Specimens were fixed with 10% neutral phosphate-buffered formalin and paraffin-embedded. All blocks were stained with hematoxylin and eosin and processed in 4μm-thick slices. The slices were stained on Benchmark XT system (Ventana, Tucson, AZ, USA) at 37°C for 16 minutes. The following antibodies were used: CD3 rabbit monoclonal antibody (mAb) clone 2GV6 (Roche); CD14 rabbit mAb clone EPR3653 (Roche); CD20 polyclonal antibody (pAb) clone L26 (Roche), Ki67 rabbit mAb clone 30–9 (Roche). No double stainings were performed.

#### Sampling

For each patient we identified in the lung the area(s) with macroscopic lesions. Then within each area we took the following samples: at least 2 from the area with the macroscopic lesions, 2 from the contiguous lung and 2 from a distant area.

The enumeration of CD20+ B-cells was performed using a +40 lens corresponding to a tissue area of 30 mm2 and immunohistochemical scores were assigned by consensus between two pathologists (FD, AB). The slides were visually estimated, and proportions of CD20 positive cells were scored and classified in 5 groups (score 1: 0–10%; score 2: 10–25%; score 3: 25–50%; score 4: 50–75%; score 5: >75% positive cells) as described [43]. The counts were performed counting the number of CD20+ cells over 100 cells for each area. Each sample was evaluated at least in 3 representative different areas. In each area the two pathologists estimated the proportion of positive cells and then the average proportions of the 3 areas was reported as the CD20-positive score. The intensity of the staining was not informative.

### Statistical analysis

Data obtained from individuals infected with Mtb, either LTBI, active TB disease or successfully treated for TB disease were compared to healthy controls using Mann-Whitney testing, with a p-value < 0.05 considered significant. However as 3 different groups were compared to the controls, multiple testing adjustments had to be made. Here we used the Kruskal-Wallis test with Dunn’s post testing and considered only those observations significant that had p < 0.05 in both the Kruskal-Wallis as well as the post test, these are indicated with a * in addition to the p-value from the Mann-Whitney test. Moreover, the effect of B-cell depletion was assessed within each group in a paired fashion using the Wilcoxon-signed-rank test, considering p < 0.05 as significant. All statistical testing was performed in GraphPad Prism version 6.02.

## Supporting Information

S1 Fig Blood cell counts and relative frequencies. Haematological characterization of blood was performed on 7 controls, 21 LTBI individuals, 23 TB patients and 25 TB treated patients. Ethnicity of donors was indicated using the following symbols: ‘black circle’ = West Europe; ‘black diamond’ = Est Europe; ‘black square’ = Africa; ‘black triangle’ = Asia; ‘black star’ = Sud America. LTBI, TB and TB treated individuals were compared to the controls using the Mann-Whitney test and a p < 0.05 was considered significant. * marks differences that remained significant after multiple test correction using Kruskal-Wallis testing with Dunn’s post-test. A. Routine haematological characterization of peripheral blood expressed as percentage of total cells present in sample. B. Absolute counts of cell subsets in whole blood.
(TIF)

S2 Fig Gating strategy B-cells. A full gating strategy compliant with MIATA [42]. A. The first step is a gate on singlets based on FSC-height and area, followed by a lymphocyte gate using FSC and SSC. Subsequently, B-cells are identified using CD20, CD21 and CD10. B. Memory B cell subsets are identified using CD27 and IgD or CD21 within a total B-cell gate. Example of B-cell proliferation using the violet tracking dye in unstimulated and anti-CD40 combined with anti- IgG/ IgM stimulated samples. C. Analysis of proliferation was combined with intracellular cytokine staining; here IL-10 on day 6 is shown.
(TIF)

S3 Fig In TB patients B-cells may localize around the granuloma. Representative histological examination of lung specimens from autopsies of patients died for pulmonary TB, scored for the B-cell number. (a) representative lung specimen with score 1; (b) representative lung specimen with score 2; (c) representative lung specimen with score 3; (d) representative lung specimen with score 4. Samples were stained with CD20 Ab. Original magnification (OM), 200x.
(TIF)

S4 Fig Gating strategy T-cells. A. The first gating step is a gate on singlets based on FSC-height and area, followed by a lymphocyte gate using FSC and SSC. Subsequently, T-cells are identified using CD3 and dead cells are excluded using the fixable viability dye Vivid. Finally, CD4^+^ and CD8^+^ T cells are separated for further downstream analysis. B. Boolean gate settings for CD25, LAG3 and FoxP3 for CD4^+^ (top row) and CD8^+^ (bottom row) T-cells in an unstimulated sample (left) and a BCG stimulated sample (right). C. Gating for inhibitory receptors PD1 (left) and KLRG1 (right) on CD4^+^ T-cells.
(TIF)

S5 Fig Memory B-cell subsets. A. Peripheral memory B-cell development following antigen specific triggering. Naïve (CD21^+^CD27^-^) B-cells differentiate in 3 different subsets, CD21^-^CD27^+^ Activated Memory B-cells that can subsequently differentiate into plasma cells; CD21^-^CD27^-^ atypical or tissue-like memory B-cells and CD21^+^CD27^+^ classical resting memory B-cells. B. Memory B-cell subsets can be differentiate using flow-cytometric analysis based on the expression of CD27 an CD21 or IgD. Comparison of these subsets relative to the healthy control population is indicate for the 3 TB infected groups, arrows indicate relative up or down regulation of specific populations.
(TIF)
