# Supplementary figures and images for: Patients with Tuberculosis Have a Dysfunctional Circulating B-Cell Compartment, Which Normalizes following Successful Treatment
Source: PLoS Pathog. 2016 Jun 15;12(6):e1005687. doi: 10.1371/journal.ppat.1005687 (PMC4909319; doi:10.1371/journal.ppat.1005687)

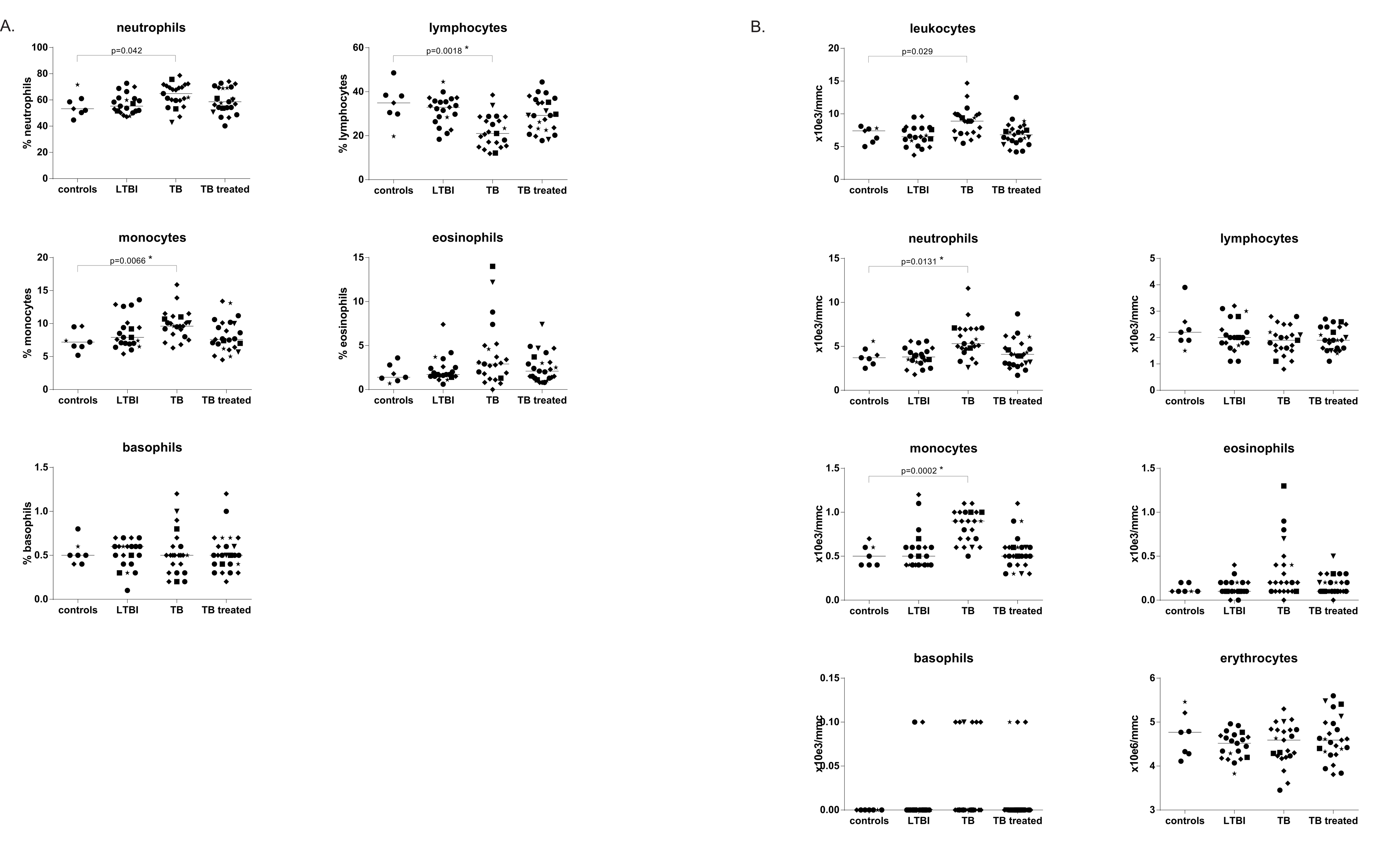

Supplement: S1 Fig — Haematological characterization of blood was performed on 7 controls, 21 LTBI individuals, 23 TB patients and 25 TB treated patients. Ethnicity of donors was indicated using the following symbols: ‘black circle’ = West Europe; ‘black diamond’ = Est Europe; ‘black square’ = Africa; ‘black triangle’ = Asia; ‘black star’ = Sud America. LTBI, TB and TB treated individuals were compared to the controls using the Mann-Whitney test and a p < 0.05 was considered significant. * marks differences that remained significant after multiple test correction using Kruskal-Wallis testing with Dunn’s post-test. A. Routine haematological characterization of peripheral blood expressed as percentage of total cells present in sample. B. Absolute counts of cell subsets in whole blood. (TIF) [file ppat.1005687.s001.tif]

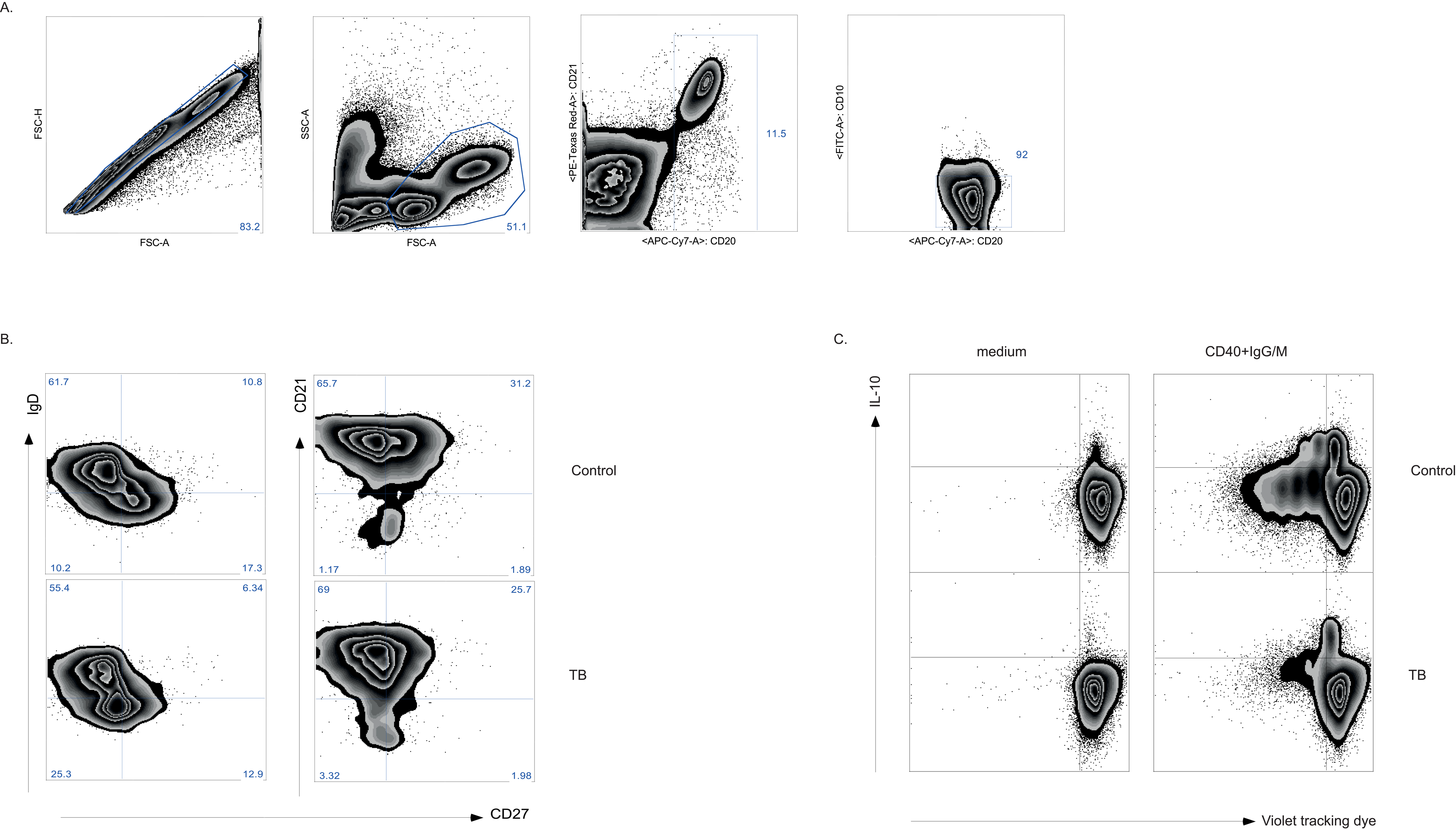

Supplement: S2 Fig — A full gating strategy compliant with MIATA [42]. A. The first step is a gate on singlets based on FSC-height and area, followed by a lymphocyte gate using FSC and SSC. Subsequently, B-cells are identified using CD20, CD21 and CD10. B. Memory B cell subsets are identified using CD27 and IgD or CD21 within a total B-cell gate. Example of B-cell proliferation using the violet tracking dye in unstimulated and anti-CD40 combined with anti- IgG/ IgM stimulated samples. C. Analysis of proliferation was combined with intracellular cytokine staining; here IL-10 on day 6 is shown. (TIF) [file ppat.1005687.s002.tif]

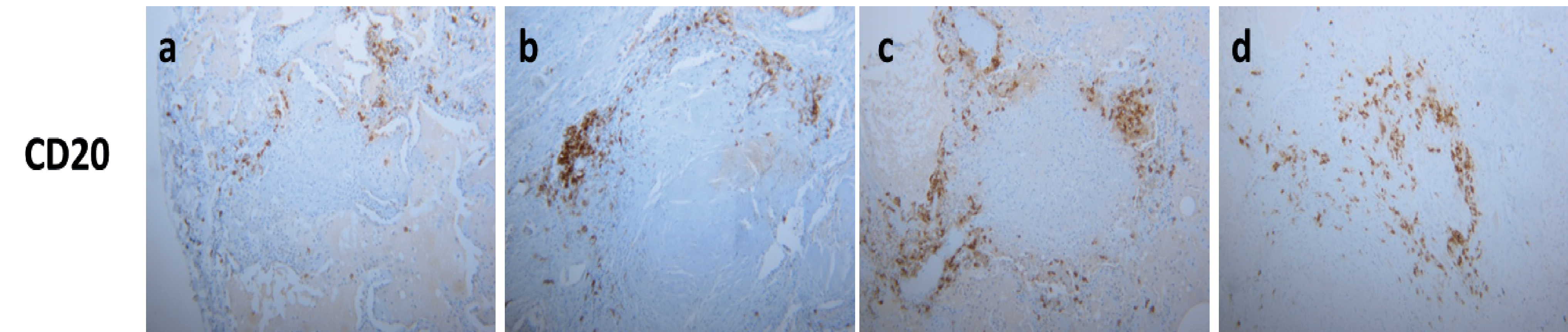

Supplement: S3 Fig — Representative histological examination of lung specimens from autopsies of patients died for pulmonary TB, scored for the B-cell number. (a) representative lung specimen with score 1; (b) representative lung specimen with score 2; (c) representative lung specimen with score 3; (d) representative lung specimen with score 4. Samples were stained with CD20 Ab. Original magnification (OM), 200x. (TIF) [file ppat.1005687.s003.tif]

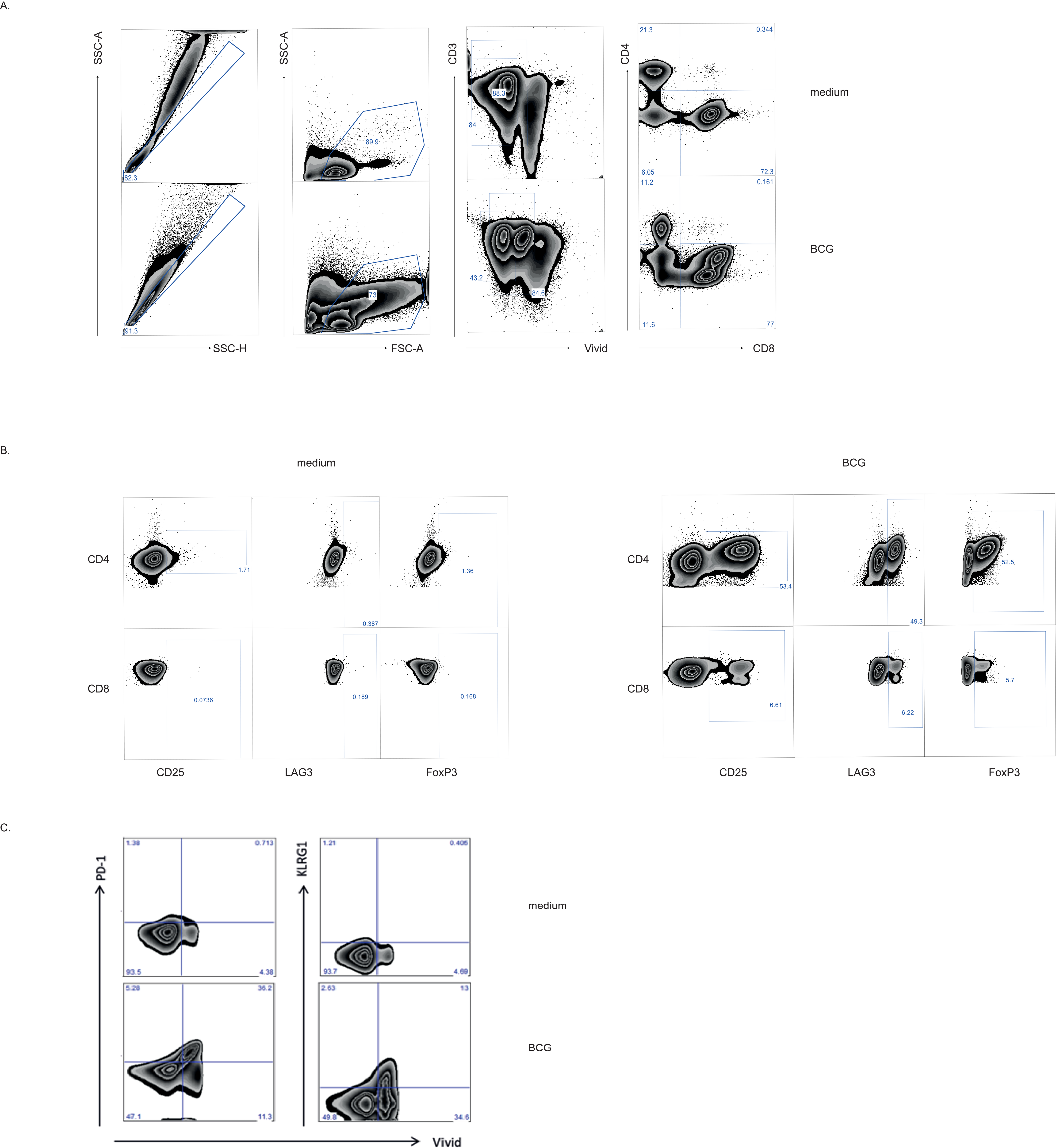

Supplement: S4 Fig — A. The first gating step is a gate on singlets based on FSC-height and area, followed by a lymphocyte gate using FSC and SSC. Subsequently, T-cells are identified using CD3 and dead cells are excluded using the fixable viability dye Vivid. Finally, CD4+ and CD8+ T cells are separated for further downstream analysis. B. Boolean gate settings for CD25, LAG3 and FoxP3 for CD4+ (top row) and CD8+ (bottom row) T-cells in an unstimulated sample (left) and a BCG stimulated sample (right). C. Gating for inhibitory receptors PD1 (left) and KLRG1 (right) on CD4+ T-cells. (TIF) [file ppat.1005687.s004.tif]

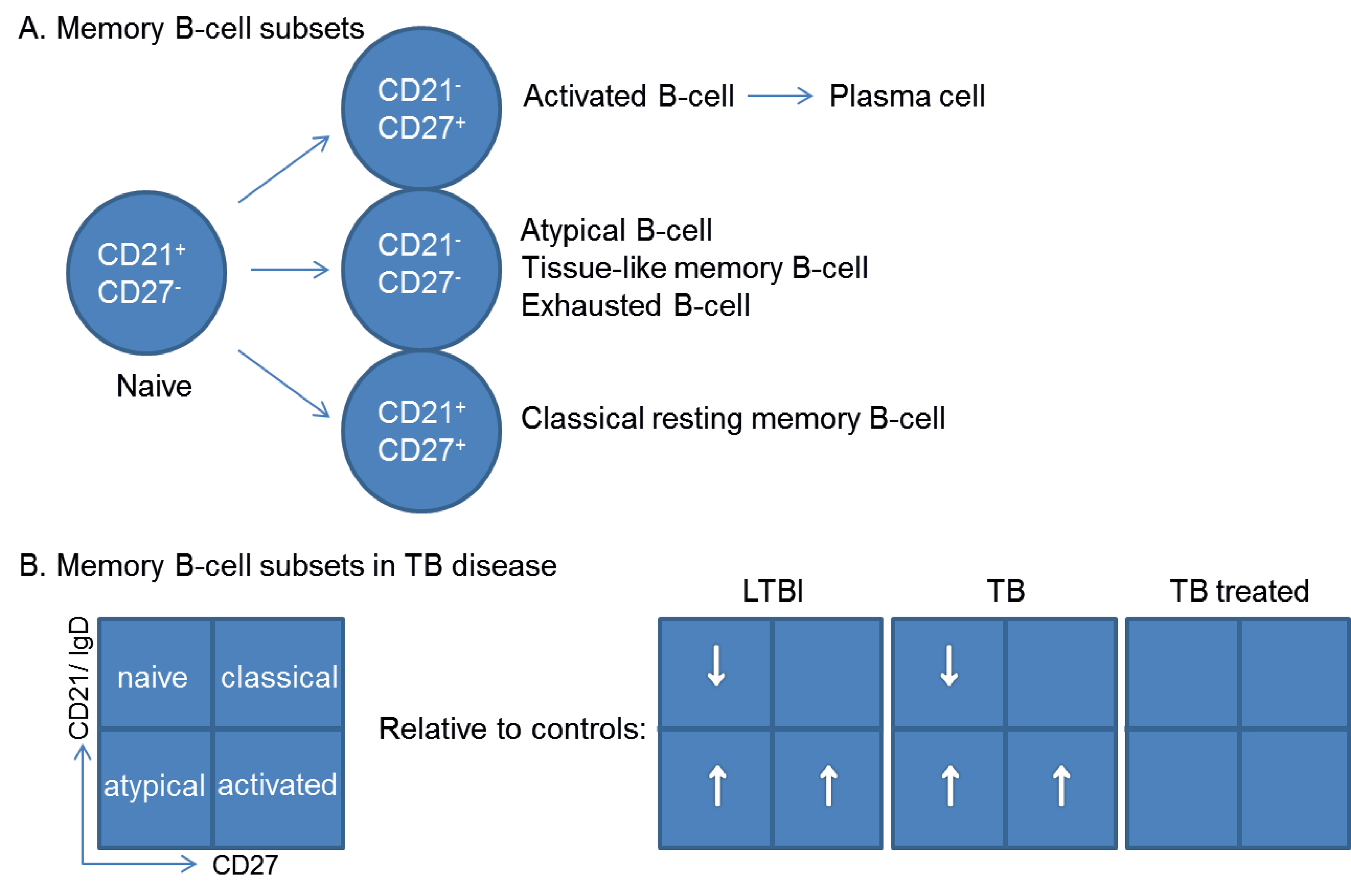

Supplement: S5 Fig — A. Peripheral memory B-cell development following antigen specific triggering. Naïve (CD21+CD27-) B-cells differentiate in 3 different subsets, CD21-CD27+ Activated Memory B-cells that can subsequently differentiate into plasma cells; CD21-CD27- atypical or tissue-like memory B-cells and CD21+CD27+ classical resting memory B-cells. B. Memory B-cell subsets can be differentiate using flow-cytometric analysis based on the expression of CD27 an CD21 or IgD. Comparison of these subsets relative to the healthy control population is indicate for the 3 TB infected groups, arrows indicate relative up or down regulation of specific populations. (TIF) [file ppat.1005687.s005.tif]
